# Optimization of Administered Activity for ^64^ Cu-SAR-bisPSMA PET Imaging in Primary Prostate Cancer: Results from a Phase I Retrospective Study (PROPELLER Sub-study)

**DOI:** 10.1055/s-0046-1824335

**Published:** 2026-06-23

**Authors:** Eva Lengyelova, Manisha Patel, Glynn Morrish, Erik Mittra, Alessandra Malaroda, Othon Gervasio, Thomas Armor

**Affiliations:** 1 Clarity Pharmaceuticals, Sydney, Australia; 2 Division of Molecular Imaging and Therapy, Oregon Health & Science University, Portland, Oregon, United States; 3 Nuclear Medicine and PET Department, Nepean Hospital, Kingswood, Australia; 4 School of Physics and Centre for Medical Radiation Physics, University of Wollongong, Wollongong, Australia

**Keywords:** ^64^ Cu-SAR-bisPSMA, PSMA, PET/CT, prostate cancer, diagnosis

## Abstract

**Background:**

^68^ Ga- and ^18^ F-labeled prostate-specific membrane antigen (PSMA)-targeted positron emission tomography (PET) agents have advanced prostate cancer (PC) imaging. ^64^ Cu-SAR-bisPSMA may offer several advantages for imaging of PC over standard-of-care PSMA PET agents due to its sarcophagine chelator, bivalent structure and longer half-life of ^64^ Cu (12.7h vs. <2h for ^68^ Ga and ^18^ F), which may lead to detection of additional and smaller lesions. This study determined the administered activity (AA) of ^64^ Cu-SAR-bisPSMA required to achieve diagnostic-quality imaging for clinical use.

**Methods:**

The PROPELLER trial (CLP03; NCT04839367) enrolled 30 men with untreated, histopathology-confirmed, primary PC with intermediate- to high-risk features. Participants received an intravenous injection of ^64^ Cu-SAR-bisPSMA at 100, 150, or 200 MBq, followed by whole-body PET/computed tomography 2 to 4 hours post-injection. Images were anonymized, randomized, and reviewed by two blinded central readers using the three-point Image Utility Classification Score. For intra-individual comparisons, a subset of images acquired at 200 MBq were digitally reconstructed to simulate 100 and 150 MBq acquisitions on the same scanner; these were randomized and then evaluated by a blinded reader. They were ranked from best image to worst image and scored with a 4-item, 5-point Likert Image Quality Score (maximum 20 points). Signal-to-noise ratio (SNR) and contrast-to-noise ratio (CNR) were also measured.

**Results:**

Mean Image Utility Classification Score was the highest in the 200 MBq ^64^ Cu-SAR-bisPSMA cohort compared with the 100 and 150 MBq cohorts (1.44, 1.17, and 0.92, respectively). For the intra-individual comparisons, images corresponding to 200 MBq were ranked as having the best image quality with a statistically significant difference in image quality ranking between AAs ( *p* = 0.0025). The mean Image Quality Score was highest at 200 MBq of ^64^ Cu-SAR-bisPSMA (16.0 ± standard deviation 1.10) versus 150 MBq (12.2 ± 1.17) and 100 MBq (10.3 ± 1.63). Quantitatively, 200 MBq of ^64^ Cu-SAR-bisPSMA had the greatest SNR in both the liver and prostate and the highest CNR for liver relative to the gluteus muscle.

**Conclusions:**

An AA of 200 MBq was identified as the optimal AA for ^64^ Cu-SAR-bisPSMA PET, yielding improved image quality across both qualitative and quantitative metrics compared with 150 and 100 MBq. These findings support the selection of 200 MBq for clinical studies evaluating ^64^ Cu-SAR-bisPSMA as an imaging agent for PC. NCT04839367, Registered 2021–04–07.

## Introduction

Prostate cancer (PC) is the second most common cancer in men. 1 Imaging for PC has conventionally included magnetic resonance imaging, computed tomography (CT), and ^99m^ Tc-methylene diphosphonate ( ^99m^ Tc-MDP) bone scans and plays an important role in diagnosis, staging, treatment planning, and disease monitoring. Unfortunately, most conventional PC imaging fails to detect small metastatic lesions, particularly in pelvic lymph nodes. 2 This has important clinical implications, both for initial staging and for localizing recurrence following definitive therapy.

Recent advances include positron emission tomography (PET) radiopharmaceuticals targeting prostate-specific membrane antigen (PSMA), such as ^68^ Ga-PSMA-11, ^18^ F-DCFPyL, and ^18^ F-rhPSMA-7.3. These have improved sensitivity and specificity for PC detection. 3 4 5 However, these agents still have limitations, including reduced sensitivity for small-volume metastases, such as those found in lymph nodes, 6 7 and logistical challenges related to the short half-lives of gallium-68 ( ^68^ Ga; 68 minutes) and fluorine-18 ( ^18^ F; 110 minutes). 8 9

Copper-64 labeled sarcophagine-bisPSMA ( ^64^ Cu-SAR-bisPSMA) offers several advantages over these agents, combining the longer half-life of ^64^ Cu (12.7 hours) with a bivalent structure, enhancing tumor uptake and retention. Preclinical imaging studies showed superior uptake and retention of ^64^ Cu-SAR-bisPSMA compared to its monomeric counterpart, 10 and in clinical studies, it demonstrated higher tumor-to-background ratios and detected more lesions than with ^68^ Ga-PSMA-11 in participants with PC. 11 The sarcophagine (SAR) chelator provides high in vivo stability with the copper isotope, increasing specificity. The longer half-life supports centralized manufacturing and broad distribution, providing logistical advantages such as flexible patient scheduling and next-day imaging.

This study sought to determine the optimal administered activity (AA) of ^64^ Cu-SAR-bisPSMA for PC PET imaging. Image quality was assessed through qualitative and quantitative approaches by nuclear medicine experts experienced in PSMA PET.

## Materials and Methods

### Study Population

The PROPELLER study (NCT04839367) was a phase I clinical trial evaluating the safety and image quality of a single AA of 100, 150, or 200 MBq of ^64^ Cu-SAR-bisPSMA in participants with PC. 11 The study was approved by the Human Research Ethics Committee, and all participants signed an informed consent form. It adhered to the ethical principles of the Declaration of Helsinki and International Council for Harmonization Good Clinical Practice.

Key inclusion criteria were adults with histopathology-confirmed, intermediate- to high-risk, untreated PC scheduled for radical prostatectomy, adequate renal function; and a ^68^ Ga-PSMA-11 PET/CT within 60 days prior to Day 0. Key exclusion criteria were prior prostatectomy, PC treatment (including androgen deprivation, radiation, or PMSA-targeted therapy), or other malignancies affecting prognosis or assessment.

### Radiopharmaceutical Preparation

^64^ Cu-SAR-bisPSMA [ ^64^ Cu][Cu(bisCOSAR-(glutamyl-D-Phe-D-Phe-8-amidooctanoyl(HO-Lys-carbonyl-Glu-OH)) _2_ )] consists of ^64^ Cu chelated by SAR linked to two PSMA inhibitors. 10 It was synthesized at South Australian Heath and Medical Research Institute, Adelaide, South Australia and Tullamarine Radiopharmacy, Melbourne, Victoria as previously described 10 and provided ready-to-use in sodium phosphate buffer with stabilizers.

### PET/CT Acquisition

^64^ Cu-SAR-bisPSMA was administered as an intravenous bolus injection within ± 10% of the prescribed activity. PET/CT was acquired 3 ± 1 hours post-injection using standard oncology protocols (3 minutes/bed position on qualified Ingenuity TF (Philips Healthcare), Biograph Vision 600 (Siemens Healthineers), Discovery MI DR (GE Healthcare), or Discovery 710 (GE Healthcare) scanners. Each scanner was qualified through phantom imaging to ensure the mean standardized uptake value (SUVmean) accuracy was within 10% of expected values.

### Inter-individual Image Quality Assessment

The images were assessed in random order by two independent, central, and trained readers blinded to participant-specific information. The readers assessed image quality using a 3-point scoring system previously developed by Delpassand et al, referred to here as the Image Utility Classification Score (IUCS), where 0 = inadequate (grainy images, poor delineation of lesions); 1 = questionable (clear images, suboptimal lesion delineation and small lesions [1 cm] hard to assess); 2 = acceptable (clear images, small and large lesion delineation). 12

### Intra-individual Image Quality Assessment

Six participants underwent PET/CT (200 MBq) at a single clinical site, using a Discovery MI DR (GE Healthcare) system in list mode, allowing retrospective reconstruction to simulate AA at 150 MBq (75% counts) and 100 MBq (50% counts). Images were reconstructed using VPFXS on a GE Advance workstation (3 iterations, 16 subsets, 6.4-mm Gaussian filter, 256 × 256 matrix). This methodology, based on count under-sampling, is a well-established technique for assessing image quality across different radiopharmaceutical AAs. 13 14 15

The qualitative assessment of image quality was performed by a blinded, independent, nuclear medicine physician experienced in PSMA PET. A set of three images of a given participant (each scan representing an equivalent to an AA of 100, 150, and 200 MBq) was displayed at the same time, side by side, in a random position order (i.e., the three images for each participant were randomized, in addition to the randomized order of each participant). The reader ranked each participant's three AA-level images from best to worst by overall visual quality. The reader also scored each image using a 4-parameter, 5-point Likert scale, the Image Quality Score (IQS; Supplementary Table S1 , available in the online version only). The IQS assessed image characteristics relevant for the diagnostic performance of PC imaging, using an adapted methodology from other image quality assessments. 16 17 The parameters included noise and reconstruction artifacts, interpretability of image data in pelvic lymph node regions, conspicuity of primary prostate lesions, and overall image quality.

### Signal-to-Noise Ratio and Contrast-to-Noise Ratio Measurements

Signal-to-noise ratio (SNR) and contrast-to-noise ratio (CNR) were measured by a certified nuclear medicine technologist using 30 cm ^3^ spherical volumes of interest (VOIs) in the liver (superior portion of segment VIII), left gluteus muscle, pelvic background (right internal iliac space), and prostate (area of greatest uptake). Measurements of SUV included the mean, maximum, and standard deviation (SD) in the sampled volume. SNR (VOI) = mean (VOI)/SD (VOI). 18 CNR = [mean (target) – mean (background)]/SD (background). 18 The independent, blinded reader assessing intra-individual image quality did not have access to the quantitative image quality results.

### Statistical Methods

For the inter-individual IUCS, the mean ± SD and 95% confidence interval (CI) were calculated for each AA cohort. IUCSs were reported separately for each reader, averaged across both readers, and averaged across all participants within each cohort. Chance-corrected inter-reader agreement was evaluated using simple kappa statistics, with corresponding standard error and 95% CI.

For the intra-individual assessments, statistical assessment of a difference in overall ranking between the different AAs was determined using a Friedman test followed by multiple pairwise comparisons using a paired signed-rank test with Bonferroni multiple testing correction. With consideration to the conservation of α-spending, given the limited available sample size and application of nonparametric testing, and the primary aim to investigate 200 MBq relative to other AAs, pairwise comparison of one group (200 MBq) with each of the other groups was undertaken. Statistical assessment of the difference in total IQS, SNR, or CNR between the different AAs was compared using a repeated-measures analysis of variance (ANOVA) followed by multiple pairwise comparisons using a paired *t* -test with Bonferroni multiple testing correction.

Quantitative data were presented as the mean ± SD. A *p* -value of less than 0.05 was considered statistically significant. Statistical analyses were conducted using SAS version 9.4 (SAS Institute Inc.) and R version 4.2.1 (R Core Team).

## Results

### Baseline Characteristics

**Fig. 1:**
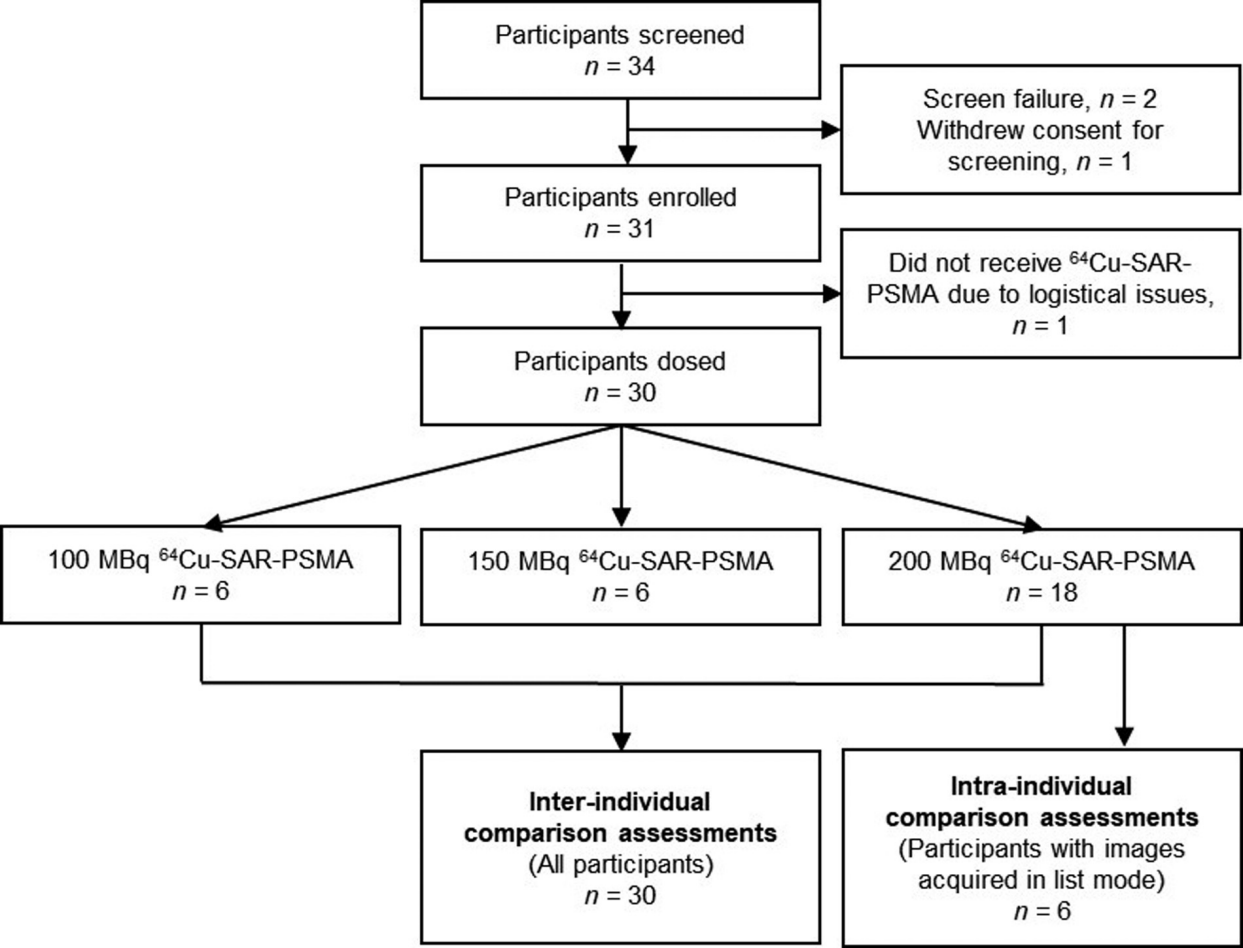
Flowchart of participant enrollment and study design. MBq, megabecquerel.

A total of 31 participants were enrolled in the study between July 13, 2021 and October 19, 2022 ( Fig. 1 ). The median participant age was 65 (range: 50–75 years). Twenty-seven out of 30 (90%) of the participants were Caucasian. Twenty-eight participants (93.3%) had acinar adenocarcinoma. The mean prostate-specific antigen level at baseline was 10.5 ng/mL (range: 1.6–36.0). Participant characteristics are shown in Supplementary Table S2 , available in the online version only.

### Administered Activities Investigated

Enrolled participants were sequentially allocated to receive a single administration of either 100 MBq ( *n* = 6), 150 MBq ( *n* = 6), or 200 MBq ( *n* = 18) of ^64^ Cu-SAR-bisPSMA followed by a whole-body PET/CT at 3 ± 1 hours post-administration ( Fig. 1 ). A subset of six participants from the 200 MBq cohort, with available list mode data, were used for intra-individual image quality comparison. This subset did not differ significantly from the overall study population.

### Inter-individual Image Utility Classification Scores

PET image quality across the three AA cohorts was evaluated by two blinded central readers using the three-point IUCS. The mean IUCS was the highest in the 200 MBq ^64^ Cu-SAR-bisPSMA cohort at 1.44 (SD: 0.54, 95% CI: 1.18–1.71) compared with the 100 and 150 MBq ^64^ Cu-SAR-bisPSMA cohorts at 1.17 (SD: 0.52, 95% CI: 0.62–1.71) and 0.92 (SD: 0.66, 95% CI: 0.22–1.61), respectively ( Table 1 ). Overall, there was a low level of agreement between the two central readers for IUCS with a kappa value of 0.06 (standard error: 0.05, 95% CI: −0.03 to 0.15).

**Table 1:** Image Utility Classification Score by administered activity and reader

^64^ Cu-SAR-bisPSMA	IUCS	Reader 1 score, *n* (%)	Reader 2 score, *n* (%)	Average IUCS (SD) [95% CI]
100 MBq *n* = 6	2	5 (83.3%)	1 (16.7%)	1.17 (0.52) [95% CI: 0.62–1.71]
	1	1 (16.7%)	1 (16.7%)	
	0	0	4 (66.7%)	
150 MBq *n* = 6	2	4 (66.7%)	1 (16.7%)	0.92 (0.66) [95% CI: 0.22–1.61]
	1	1 (16.7%)	0	
	0	1 (16.7%)	5 (83.3%)	
200 MBq *n* = 18	2	16 (88.9%)	7 (38.9%)	1.44 (0.54) [95% CI: 1.18–1.71]
	1	2 (11.1%)	4 (22.2%)	
	0	0	7 (38.9%)	

Abbreviations: CI, confidence interval; IUCS, Image Utility Classification Score; MBq, megabecquerel; SD, standard deviation.

Note: An IUCS of 2 is acceptable, a score of 1 is questionable, and a score of 0 is inadequate.

### Intra-individual Image Rankings

**Fig. 2:**
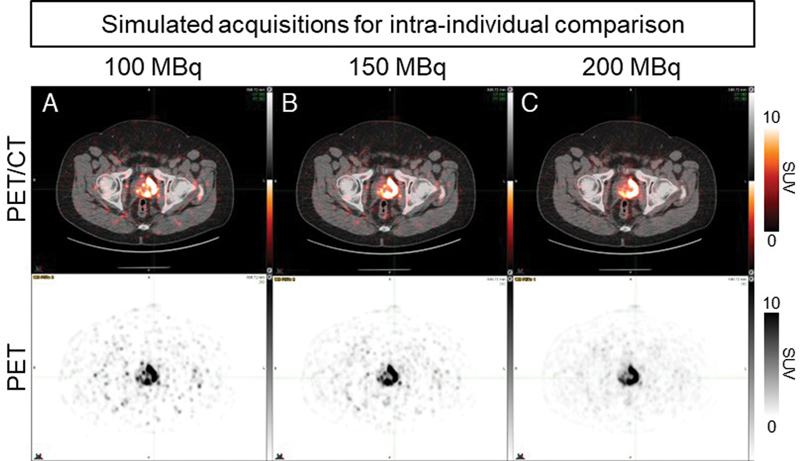
Representative PET/CT (top row) or PET only (bottom row) images for 100 ( **A** ), 150 ( **B** ), and 200 ( **C** ) MBq of ^64^ Cu-SAR-bisPSMA used for intra-individual comparison studies. Substantial ^64^ Cu-SAR-bisPSMA uptake was seen in the prostate across all administered activities with increased background/graininess at lower equivalent administered activities (A, B). SUV ranges from 0 to 10. Image quality was limited due to the age of the scanner used for image capture. CT, computed tomography; MBq, megabecquerel; PET, positron emission tomography; SUV, standardized uptake value.

In six participants, we next conducted intra-individual comparisons of PET images using 200 MBq ^64^ Cu-SAR-bisPSMA PET acquired in list mode, which allows for post-acquisition reconstruction of images as if they were acquired using 100 or 150 MBq ( Fig. 2 ). 13 14 15 The scans representing different AAs were ranked in order of best image quality. The 200 MBq images were ranked for all six participants as having the best overall visual quality, followed by the 150 MBq images, and then the 100 MBq images ( Table 2 ; difference between ranking across all AAs, *p* = 0.002479). The ranking of the 200 MBq images above both the 150 and 100 MBq images was shown to be statistically significant ( *p* = 0.039 for each comparison).

**Table 2:** Ranking of images of different administered activities by image quality

Administered activity ( *n* )	Rank position for image quality		
	Ranked 1st (best), *n* (%)	Ranked 2nd, *n* (%)	Ranked 3rd (worst), *n* (%)
100 MBq ( *n* = 6)	0 (0.0%)	0 (0.0%)	6 (100.0%)
150 MBq ( *n* = 6)	0 (0.0%)	6 (100.0%)	0 (0.0%)
200 MBq ( *n* = 6)	6 (100.0%)	0 (0.0%)	0 (0.0%)
	***p*** ** -Value a**		
All groups ( *n* = 18, 6 per group)	0.002479		
200 vs. 150 MBq ( *n* = 12, 6 per group)	0.0393		
200 vs. 100 MBq ( *n* = 12, 6 per group)	0.0393		

Abbreviation: MBq, megabecquerel.

Note: Here, n denotes the number of images in each dose cohort. For intra-individual image quality comparisons, six participants with list mode data in the 200 MBq group were used. Their 200 MBq images were reconstructed to simulate acquisitions at 100 MBq and 150 MBq, enabling direct intra-individual comparison of image quality across dose levels.

a For statistical test, the Friedman rank sum test was used for comparison of all groups, and the Wilcoxon signed rank test with Bonferroni correction was used for pairwise comparison for individual groups. Adjusted *p* -value presented.

### Intra-individual Image Quality Scores

To assess scan characteristics relevant for the diagnostic performance of imaging PC, each image was scored using the IQS ( Supplementary Table S1 , available in the online version only). Images at 200 MBq had a significantly higher mean total IQS (16.0 ± SD 1.10) compared with 150 MBq (12.2 ± 1.17, *p* = 0.00018) or 100 MBq (10.3 ± 1.63, *p* = 0.00076; all group comparison *p* ≤ 0.0001) ( Table 3 ). The mean total IQS at 150 MBq was also significantly higher than at 100 MBq (12.2 ± 1.17 vs. 10.3 ± 1.63, *p* = 0.00568). Images were also assessed within each parameter of the IQS ( Supplementary Table S3 , available in the online version only). Within each parameter (noise and reconstruction artifacts, interpretability of image data in pelvic lymph node regions, conspicuity of primary prostate lesions, and overall image quality), the 200 MBq image had the highest mean score compared with the 150 and 100 MBq images. Notably, in the parameter of noise and reconstruction artifacts, the reader deemed all scans at 200 MBq to be of typical or comparable quality to standard clinical PET imaging, whereas the lower AAs levels yielded images with noise greater than standard PET, which, in some cases, would make interpretation difficult. For the interpretability of the pelvic lymph node regions, which directly impacts the clinical utility of PSMA PET, the reader deemed all scans at 200 MBq to be of adequate quality to allow interpretation. In contrast, scans at 100 and 150 MBq were deemed of poor, barely interpretable quality for the assessment of the pelvic lymph node regions. In one case, the increased noise found in a 100 MBq equivalent image enhanced image artifacts to appear as if there was uptake in a lymph node ( Supplementary Fig. S1 , available in the online version only); these artifacts were not observed in the images of the same individual at higher AA equivalent images.

**Table 3:** Total Image Quality Score by administered activity

Statistic	Total IQS		
	100 MBq ( *n* = 6)	150 MBq ( *n* = 6)	200 MBq ( *n* = 6)
Mean ± SD	10.3 ± 1.63	12.2 ± 1.17	16.0 ± 1.10
Median (range)	10.0 (9–13)	12.0 (11–14)	16.0 (15–17)

Abbreviations: IQS, Image Quality Score; MBq, megabecquerel; SD, standard deviation.

Note: Images at 200 MBq had a significantly higher mean total IQS compared with 150 MBq ( *p* = 0.00018) or 100 MBq ( *p* = 0.00076; all group comparison *p* ≤ 0.0001).

### Intra-individual Quantitative Analysis of Signal-to-Noise Ratio and Contrast-to-Noise Ratio

To further support the qualitative image quality assessments, we also investigated quantitative differences using two objective metrics: SNR and CNR. High SNR and CNR are established parameters of PET image quality and correlate with improved diagnostic performance. 18 SNR was assessed in both the prostate and liver, with the liver serving as a physiologic reference region. The images at 200 MBq had the highest mean SNR in the prostate (1.571 ± 0.5438) compared with images at an equivalent AA of 150 MBq (1.341 ± 0.4180; *p* = 0.031) or 100 MBq (1.288 ± 0.3624; *p* = 0.061), with all *p* -values adjusted using Bonferroni correction ( Table 4 ). No statistically significant difference was observed between the 150 and 100 MBq levels ( *p* = 0.412). Similarly, liver SNR was highest at 200 MBq (4.649 ± 1.243) compared with 150 MBq (3.221 ± 0.6878; *p* = 0.018) or 100 MBq (2.676 ± 0.5732; *p* = 0.011). A significant difference was seen between 150 and 100 MBq ( *p* = 0.006).

**Table 4:** Summary of signal-to-noise ratio and contrast-to-noise ratio by location and administered activity

Location	Statistic	100 MBq ( *n* = 6)	150 MBq ( *n* = 6)	200 MBq ( *n* = 6)
**SNR**				
** Prostate**	Mean ± SD	1.288 ± 0.362	1.341 ± 0.418	1.571 ± 0.544
	Median (range)	1.341 (0.60–1.69)	1.375 (0.59–1.88)	1.647 (0.63–2.30)
** Liver**	Mean ± SD	2.676 ± 0.573	3.221 ± 0.688	4.649 ± 1.243
	Median (range)	2.697 (2.05–3.60)	3.094 (2.60–4.36)	4.369 (3.05–6.16)
**CNR**				
** Prostate/pelvic background**	Mean ± SD	9.174 ± 11.278	10.807 ± 12.988	16.497 ± 22.411
	Median (range)	4.921 (1.96–31.52)	5.575 (2.13–36.28)	7.568 (2.70–61.31)
** Liver/gluteus muscle**	Mean ± SD	11.74 ± 7.371	14.124 ± 8.016	21.504 ± 11.23
	Median (range)	8.866 (7.09–16.52)	10.37 (9.65–30.01)	16.775 (14.88–43.86)

Abbreviations: CNR, contrast-to-noise ratio; MBq, megabecquerel; SD, standard deviation; SNR, signal-to-noise ratio.

CNR was assessed in the prostate relative to the surrounding pelvic background, and in the liver relative to adjacent muscle. Although the images at 200 MBq had the highest mean prostate CNR (16.497 ± 22.4112) compared with images at an equivalent AA of 150 MBq (10.807 ± 12.9878; *p* = 0.61) or 100 MBq (9.174 ± 11.2780; *p* = 0.51), the differences were not statistically significant, likely due to high inter-individual variability ( Table 4 ). Similarly, there was no statistical difference in the prostate CNR between the images at an equivalent AA of 150 or 100 MBq ( *p* = 0.23). The image at 200 MBq had the highest mean liver CNR (21.504 ± 11.2303) compared with images at an equivalent AA of 150 MBq (14.124 ± 8.0159; *p* = 0.0095) or 100 MBq (11.740 ± 7.3706; *p* = 0.0070), with a significant difference also observed between 150 and 100 MBq ( *p* = 0.0064).

## Discussion

^64^ Cu-SAR-bisPSMA is an emerging radiotracer that may enhance PSMA-targeted PET by improved and delayed imaging, due to its longer isotope half-life, two PSMA-targeting groups, and SAR chelator. 19 Additionally, it can be used in a theranostic setting with copper-67 for therapy. As such, it is critical to define the optimal AA of ^64^ Cu-SAR-bisPSMA to support its safe and effective use in clinical practice. Leveraging novel methodology and complementary approaches, this pilot study established 200 MBq of ^64^ Cu-SAR-bisPSMA as the optimal AA for PC imaging. Inter-individual analysis demonstrated that 200 MBq of ^64^ Cu-SAR-bisPSMA provided the highest IUCS when compared with AAs of 100 and 150 MBq. In intra-individual studies, 200 MBq images were consistently ranked by blinded review as having the best overall quality compared with images using equivalent AAs at 150 and 100 MBq, as well as demonstrating better scoring in key image quality parameters related to PET. Lastly, objective and quantifiable assessments of image quality, SNR and CNR, supported these results. The 200 MBq of ^64^ Cu-SAR-bisPSMA images had the highest SNR for the prostate and liver compared with equivalent AAs at 150 and 100 MBq. Similarly, 200 MBq of ^64^ Cu-SAR-bisPSMA PET images had the highest CNR for the target liver versus muscle background compared with equivalent AAs at 150 and 100 MBq.

In addition to providing the highest total IQS and highest SNR and CNR values, 200 MBq of ^64^ Cu-SAR-bisPSMA had less background noise or graininess compared with lower AAs, especially at 100 MBq. Noisy or grainy image background can limit and interfere with lesion interpretation, particularly the assessment of pelvic lymph node involvement, where lesions are often very small. For example, the increased noise found in one of the 100 MBq equivalent images resulted in a reconstruction artifact that mimicked the appearance of a lymph node lesion. This was not observed on the 150 and 200 MBq images of the same participant. Image quality is important as incorrect diagnosis (e.g., false-positive or false-negative results) can have important consequences in patient management. False-positive findings could lead to unnecessary biopsies, surgeries, or other interventions, exposing patients to avoidable risks and complications. Likewise, a false-negative finding could lead to tumor under-staging and subsequent changes in therapeutic decision-making, especially for metastatic disease. Furthermore, the estimated whole-body effective radiation doses of 100-200 MBq ^64^ Cu-SAR-bisPSMA (unpublished data) fall within the range typically considered to be of low-risk. ^20^ In this context, 200 MBq ^64^ Cu-SAR-bisPSMA PET/CT achieves a favourable balance between image quality and radiation exposure supporting its use as the optimal AA for ^64^ Cu-SAR-bisPSMA PET.

Diagnostic radiopharmaceuticals targeting PSMA have become increasingly important for PC imaging due to their improved sensitivity and specificity compared with conventional imaging. ^64^ Cu-SAR-bisPSMA may allow improved and delayed imaging due to its key components: (1) ^64^ Cu isotope, and (2) SAR, a bifunctional metal chelator. First, the longer half-life of ^64^ Cu (12.7 hours), compared with ^68^ Ga (68 minutes) and ^18^ F (110 minutes), 19 contributes toward longer imaging windows. Later image acquisition times permit additional tumor uptake and also clearance of nonspecific background activity, which in turn improves tumor-to-background ratios. This is important for identifying smaller lesions, which may not be possible with other tracers. 6 It also allows more flexibility for patient scheduling and broader isotope distribution to clinical sites from the production facility. Second, the two PSMA-targeting groups improve tumor uptake and retention. 10 Lastly, the SAR chelator in ^64^ Cu-SAR-bisPSMA is highly stable and prevents copper leakage into the body, 10 21 enabling better retention for improved diagnostic and therapeutic applications.

There were several innovations in this study. First, we utilized a novel methodology that enabled evaluation of image quality across different AAs within the same participant, eliminating inter-individual variability, thus allowing for a more efficient comparison of image quality performance of different AAs. This type of direct comparison would not be feasible in a real-world study, as it would subject participants to multiple tracer injections and PET scans within a small time frame. Second, this study also used a new image quality metric, the IQS, to assess scan characteristics that are highly relevant for imaging PC, using a methodology adapted from other image quality assessments. 16 17 22 23 24 The IQS improves upon previous image quality assessment tools as it is tailored for parameters important for PET imaging of PC and its clinical interpretation, as evidenced by parameters that focus on primary prostate lesions, as well as pelvic lymph nodes. Furthermore, the scoring range was sufficient to allow differentiation between the image quality of different AAs.

There were several limitations to this study. First, the small sample size limited the analysis of the performance of different AAs of ^64^ Cu-SAR-bisPSMA. Second, although the qualitative studies were blinded and images randomized, the visual interpretation was still subjective to the reader. To help control for this, the readers were well-trained and familiar with reading PSMA PET. Third, the intra-individual studies were performed by a single blinded reviewer; although this individual was well-versed in interpreting PSMA PET, it is possible that other reviewers may have scored the scans differently. Lastly, this study was restricted to a single imaging acquisition time point at 2 to 4 hours post-injection. Additional studies are underway to further elucidate the clinical utility of ^64^ Cu-SAR-bisPSMA as an imaging agent for PC (NCT06056830 and NCT06970847).

## Conclusion

In summary, 200 MBq of ^64^ Cu-SAR-bisPSMA had the highest total IUCS, IQS, SNR, and CNR values compared with images using equivalent AAs of 100 and 150 MBq. Images at 200 MBq were consistently ranked by blinded review as having the best overall quality compared with images using equivalent AAs of 100 and 150 MBq, as well as demonstrating better scoring in key image quality parameters related to PET. These results support using 200 MBq of ^64^ Cu-SAR-bisPSMA for PC imaging.
