# Supplementary material for: Optimization of Administered Activity for 64 Cu-SAR-bisPSMA PET Imaging in Primary Prostate Cancer: Results from a Phase I Retrospective Study (PROPELLER Sub-study)
Source: World J Nucl Med. 2026 Jun 23;25(2):107–14. doi: 10.1055/s-0046-1824335 (PMC13327745; doi:10.1055/s-0046-1824335)
Supplement: Supplementary file 1 — Supplementary Material [file 10-1055-s-0046-1824335-s2620007.pdf]

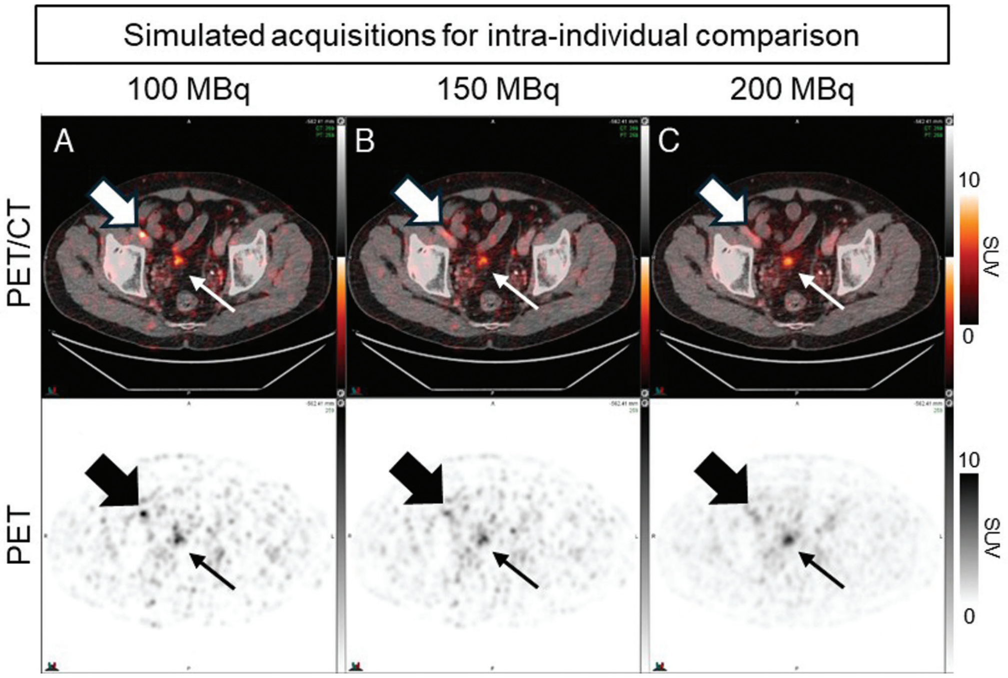

Supplementary Fig. S1 PET/CT (top row) or PET only (bottom row) images for 100 **(A)**, 150 **(B)**, and 200 **(C)** MBq of ^{64}Cu-SAR-bisPSMA. Noise from a low-count PET emission image (A, 100 MBq) mimicking lymph node uptake (thick arrow), which is not evident on the same image within the same participant with twice the activity (C, 200 MBq). In comparison, uptake in the prostate (thin arrow) is present on images for 100, 150, and 200 MBq of ^{64}Cu-SAR-bisPSMA. Image quality was limited due to the age of the scanner used for image capture. SUV ranges from 0 to 10. CT, computed tomography; MBq, megabecquerel; PET, positron emission tomography; SUV, standardized uptake value.

Supplementary Table S1 Image Quality Score (IQS) parameters and 5-point Likert scale

Image quality parameters	5-point Likert scale				
	1	2	3	4	5
Noise and reconstruction artifact	High or excessive level, hindering or preventing interpretation	Moderate or substantial, making interpretation difficult	Greater than standard PET imaging, but does not hinder interpretation	Typical/comparable to standard clinical PET imaging	Minimal or near-imperceptible
Interpretability of image data in pelvic LN regions	Nondiagnostic unable to assess	Nondiagnostic partially interpretable	Diagnostic: poor, barely interpretable	Diagnostic: adequate, interpretable	Diagnostic: good/excellent, clearly interpretable
Conspicuity of primary prostate lesions	No discernable contrast	Discernible contrast, poor visibility	Moderate contrast and uptake	Good contrast and uptake	Excellent contrast and uptake
Overall image quality	Unacceptable	Poor	Acceptable	Good	Excellent

Abbreviations: IQS, Image Quality Score; LN, lymph node; PET, positron emission tomography.

 © 2026. The Author(s).

Supplementary Table S2 Demographic and baseline characteristics

Variable	All participants ($n = 30$)	Intra-individual comparison study ($n = 6$)
Age (years), mean (range)	65 (50–75)	64.3 (50–73)
Self-reported race, *n* (%)		
Native Hawaiian or Other Pacific Islander	1 (3.3%)	
Caucasian	27 (90.0%)	
Other	2 (6.7%)	
Tumor type, *n* (%)		
Acinar adenocarcinoma	28 (93.3%)	6 (100.0%)
Ductal adenocarcinoma	2 (6.7%)	0
Tumor stage, *n* (%)		
Stage IIb	1 (3.3%)	0
Stage IIc	15 (50.0%)	4 (66.7%)
Stage IIIa	3 (10.0%)	0
Stage IIIb	1 (3.3%)	0
Stage IIIc	8 (26.7%)	2 (33.3%)
ISUP grade group, *n* (%)		
Grade group 2	3 (10.0%)	0
Grade group 3	12 (40.0%)	2 (33.3%)
Grade group 4	7 (23.3%)	2 (33.3%)
Grade group 5	8 (26.7%)	2 (33.3%)
PSA level, mean (range) ng/mL	10.5 (1.6–36.0)	8.6 (3.9–19.1)

Abbreviations: ISUP, International Society of Urological Pathologists; PSA, prostate-specific antigen; ng/mL, nanogram per milliliter.

Supplementary Table S3 Image Quality Score (IQS) frequency by individual parameters and administered activity

Parameter	Administered activity	Image Quality Score (IQS) (higher score represents higher quality)				
		1	2	3	4	5
		n (%)	*n* (%)	*n* (%)	*n* (%)	*n* (%)
**Noise and reconstruction artifact**	200 MBq	0	0	0	6 (100%)	0
	150 MBq	0	0	6 (100%)	0	0
	100 MBq	0	4 (66.7%)	2 (33.3%)	0	0
**Interpretability of image data in pelvic lymph node regions**	200 MBq	0	0	0	6 (100%)	0
	150 MBq	0	0	6 (100%)	0	0
	100 MBq	0	1 (16.7%)	5 (83.3%)	0	0
**Conspicuity of primary prostate lesions**	200 MBq	0	0	3 (50.0%)	0	3 (50.0%)
	150 MBq	0	2 (33.3%)	2 (33.3%)	1 (16.7%)	1 (16.7%)
	100 MBq	0	2 (33.3%)	2 (33.3%)	1 (16.7%)	1 (16.7%)
**Overall image quality**	200 MBq	0	0	0	6 (100%)	0
	150 MBq	0	0	6 (100%)	0	0
	100 MBq	0	6 (100%)	0	0	0

Note: Add: The number of images assessed in each dose cohort was 6.
Abbreviations: IQS, Image Quality Score; MBq, megabecquerel.

 © 2026. The Author(s).
